# Evaluation of Antidiabetic and Antihyperlipidemic Effects of *Peganum harmala* Seeds in Diabetic Rats

**DOI:** 10.1155/2016/7389864

**Published:** 2016-04-14

**Authors:** Gholamreza Komeili, Mohammad Hashemi, Mohsen Bameri-Niafar

**Affiliations:** ^1^Department of Physiology, School of Medicine, Zahedan University of Medical Sciences, Zahedan 98167-43181, Iran; ^2^Department of Clinical Biochemistry, School of Medicine, Zahedan University of Medical Sciences, Zahedan 98167-43181, Iran

## Abstract

The present study was carried out to investigate the antidiabetic and antihyperlipidemic properties of hydroalcoholic extract of* Peganum harmala* in streptozotocin-induced diabetic male rats. In an experimental study, 64 normal Wistar albino male rats (200–230 g) were randomly divided into 8 groups. Control and diabetic rats were treated with normal saline and three different doses (30, 60, and 120 mg/kg) of hydroalcoholic extract of* Peganum harmala* seeds for 4 weeks orally. At the end of treatment, blood samples were taken and glucose, triglycerides, total cholesterol, LDL-c, HDL-c, malondialdehyde (MDA), total antioxidant capacity (TCA), ALT, AST, GGT, bilirubin, and glycosylated hemoglobin (HbA_1C_) were determined. STZ-induced diabetic rats showed significant changes in the values of glucose, triglycerides, total cholesterol, LDL-c, MDA, TAC, ALT, AST, GGT, bilirubin, and HbA_1C_ in comparison with normal rats. Administration of the extract to diabetic rats resulted in a remarkable decrease in glucose, lipid profiles, MDA, ALT, AST, GGT, bilirubin, and HbA_1C_ levels and increase in TAC relative to diabetic group. The results of this study indicated that hydroalcoholic extract of* Peganum harmala* seeds possesses antidiabetic and hypolipidemic activities and could be useful in treatment of diabetes.

## 1. Introduction

Diabetes mellitus (DM) is a group of metabolic disorders characterized by circulating hyperglycemia resulting from either defect in insulin secretion or insulin action. According to the international diabetes federation (IDF), it was estimated that more than 382 million people throughout the world had diabetes in 2013. Its prevalence is increasing rapidly, particularly in developing countries, and is expected to rise to 552 million by 2030 [1]. According to the latest evaluations of the World Health Organization (WHO), the global prevalence of diabetes in adult was 9% in 2014. In 2012 diabetes was the direct cause of 1.5 million deaths. More than 80% of diabetes deaths occur in low- and middle-income countries [2]. Subjects with diabetes have an increased risk of developing a number of serious health problems. Consistently high blood glucose levels can lead to serious diseases affecting the heart and blood vessels, eyes, kidneys, nerves, and teeth period. In addition, people with DM also have a higher risk of developing infections.

Various treatment options are available such as lifestyle modifications, proper nutritional management, and oral medication [3].

Animal models have been used generally for assessment of the etiology of diabetes complications. The pathogenesis of DM and its complications are associated with the overproduction of reactive oxygen species (ROS) and reduction of the endogenous antioxidant system (enzymatic and nonenzymatic), leading to oxidative stress [4]. Streptozotocin (STZ) is the most commonly used agent to induce experimental diabetes model [5]. The antidiabetogenic, cholesterol-lowering, anti-inflammatory, and other metabolically beneficial qualities of phytochemicals from various plant extracts have been evaluated in a number of animal and human studies [6]. Iran is fortunate to have such a varied climate in which virtually approximately any medicinal plant can grow. The use of medicinal plants has increased as an alternative for the treatment of diabetes because modern medicines are identified with several side effects and are also expensive [7].* Peganum harmala* is customarily and usually used for medicinal purposes since ancient times [8]. This plant is widely scattered and used as medicinal plant in central Asia, North Africa, and Middle East [9]. The root and seeds enclose several alkaloids that are pharmacologically active and accountable for their effects. Phytochemical studies of* Peganum harmala* led to the isolation of different types of chemical ingredients such as alkaloids, steroids, and flavonoids from seeds, leaves, flowers, stems, and roots [10].* Peganum harmala* is rich in alkaloids of *β*-carbolines derivatives [11]. These alkaloids have a broad spectrum of potent therapeutic activities such as anticancer [12, 13], analgesic, and antinociceptive [14]. It has been shown that* Peganum harmala* has hypoglycemic and cytoprotective effects [7, 15].

In this study, we aimed to evaluate the effect of hydroalcoholic extract of* Peganum harmala *on blood glucose, lipid profile, liver function tests, and malondialdehyde (MDA) and total antioxidant capacity (TAC) in diabetic rats.

## 2. Materials and Methods

### 2.1. Rats

Streptozotocin (STZ) was obtained from Sigma Chemical Company. All experimental procedures were approved by local ethics committee of Zahedan University of Medical Sciences. 64 normal adult male Wistar albino rats weighing 200–230 g were chosen and housed in single cages under standard laboratory conditions (temperature 23 ± 2°C, 12 h light and dark cycle) for one week aimed at acclimatization [16]. Animals are fed with standard pellet diet and water* ad libitum*.

Then rats were allocated in 8 groups randomly (8 in each group). These groups include intact group, three control groups that received daily 30, 60, and 120 mg/kg extract of* Peganum harmala*, respectively, diabetic group, and three diabetic groups that received 30, 60, and 120 mg/kg extract of* Peganum harmala*, respectively. Duration of experiments was four weeks and water and food intake measured daily. After emphasis of diabetes, animals were treated with hydroalcoholic extract of* Peganum harmala* seeds at 30, 60, and 120 mg/kg orally for four weeks. All experiments started at 9:00 every day.

### 2.2. Preparation of Hydroalcoholic Extract of* Peganum harmala*

The seeds of* Peganum harmala* were collected from Zahedan in 2014. Plant was identified and authenticated by Botany Department of Sistan and Baluchestan University. Powdered seed of* Peganum harmala* (20 g) was placed in Soxhlet system with ethanol (70%) for 8 hours. Then extract dried in incubator (37°C) to give a final yield of approximately 10% hydroalcoholic extract of seeds. In time of experiments, the extract was dissolved in normal saline and animals were force-fed with above-mentioned doses.

### 2.3. Induction of Diabetes

Overnight fasted animals were made diabetic by intravenous injection of streptozotocin (STZ) at 50 mg/kg of body weights [17]. Blood glucose (BG) level was measured after 72 hours and animals showing BG level over 250 mg/dL were considered diabetic [18]. At the end of experiments, animals were killed by decapitation after overnight fasting and blood samples were taken in tubes with and without anticoagulant. Biochemical parameters such as blood glucose, triglyceride, total cholesterol, HDL-c, alanine aminotransferase (ALT), aspartate aminotransferase (AST), gamma glutamyl transferase (GGT), and bilirubin were determined using commercial available kits (Pars Azmoon, Tehran) by an automatic biochemistry analyzer (T 1500, Italy). LDL-c value was calculated by Friedewald equation. HbA_1C_ values were measured using commercial available kit (Pishtazteb, Iran).

Serum malondialdehyde (MDA) level was measured as the end product of lipid peroxidation by the thiobarbituric acid (TBA) reaction method as described previously [19]. Total antioxidant capacity was measured with ferric reducing ability of plasma (FRAP) method as described previously [20, 21].

### 2.4. Statistical Analysis

Data are presented as mean ± SD. Statistical analysis was done by SPSS software version 17 (SPSS, Inc., Chicago, Il, USA). Comparison of the data between groups was performed using one-way analysis of variance (ANOVA) followed by Tukey's post hoc test. *P* < 0.05 were considered statistically significant.

## 3. Results

Our findings indicated that induction of T1DM causes the increased food and water intake and decrease in body weight significantly compared to intact group (*P* < 0.01). As shown in Table 1, consumption of extracts in nondiabetic and diabetic animals did not significantly have an effect on body weight to water and food intake (*P* > 0.05). There is no significant difference in body weight and food and water intake at start of experiments (*P* > 0.05).

Also, the data showed that diabetes induction causes a significant increase in glucose, triglyceride, total cholesterol, and LDL-c (*P* < 0.01) and decrease in HDL-c (*P* < 0.05), whereas administration of* Peganum harmala* causes significant decrease in glucose (in all doses, *P* < 0.001), total cholesterol (in doses of 60 and 120 mg/kg, *P* < 0.01), and LDL-c (in all doses of extract, *P* < 0.01), increase in HDL-c (in all doses of extract, *P* < 0.01) in comparison with diabetic group (Table 2), and no effect on triglyceride level.

**Figure 1:**
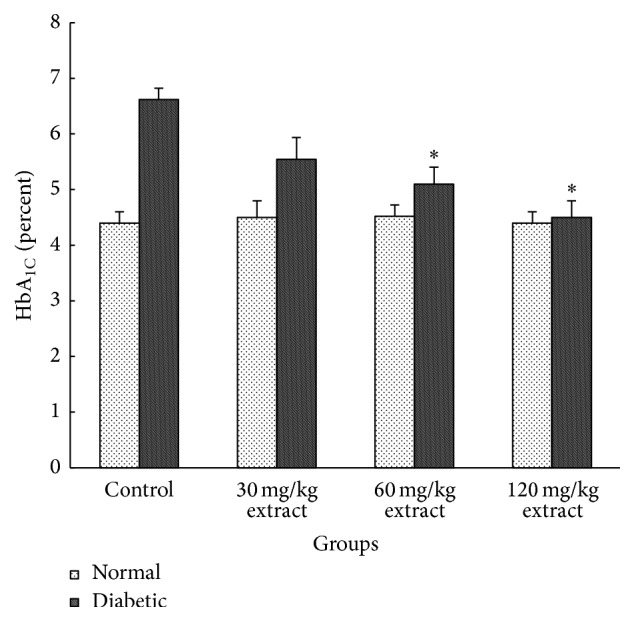
The mean value of HbA_1C_ in all different groups (data are shown in mean ± SEM, *n* = 8). ^*∗*^ *P* < 0.01 in comparison with control diabetic group.

Figure 1 shows that HbA_1C_ increased significantly in diabetic animals (*P* < 0.001) and consumption of extract decreases the HbA_1C_ in doses of 60 mg/kg (*P* < 0.01) and 120 mg/kg (*P* < 0.001) compared with diabetic group.

**Figure 2:**
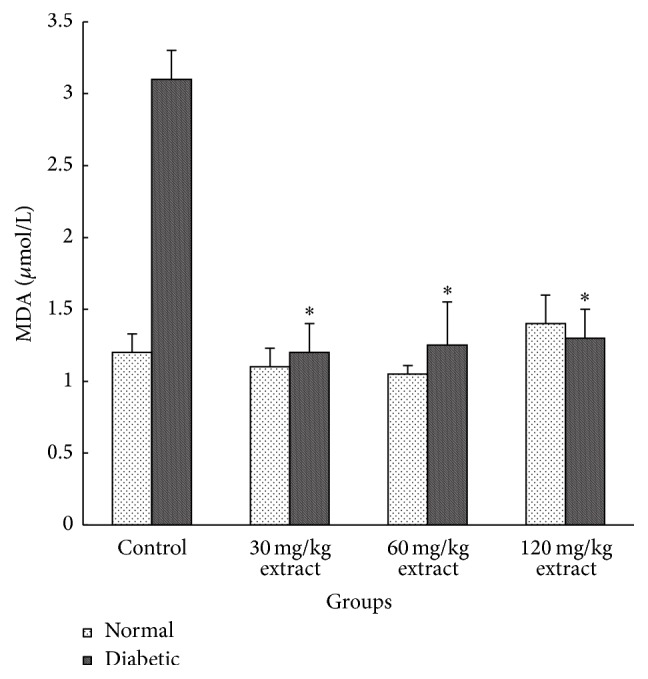
Mean values of malondialdehyde (MDA) in different groups (data shown as mean ± SEM, *n* = 8). Extract consumption decreased the levels of MDA in comparison with control diabetic group. ^*∗*^ *P* < 0.01 in comparison with control diabetic group.

**Figure 3:**
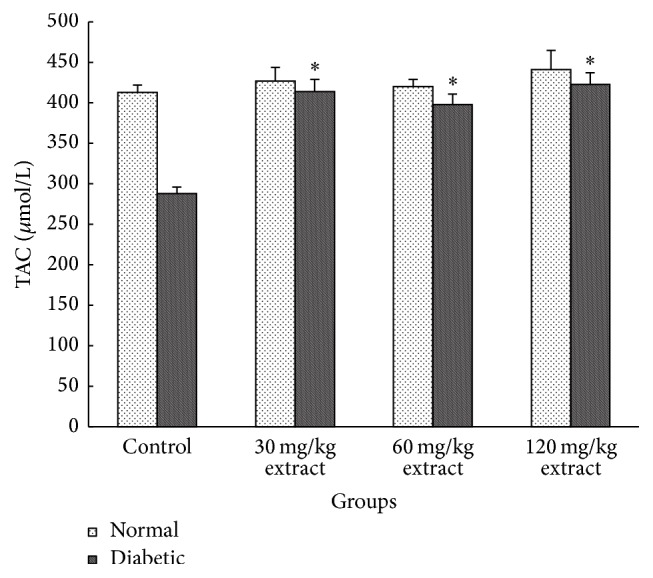
Mean values of total antioxidant capacity (TAC) in different groups. Extract consumption increased TAC in all doses (data shown as mean ± SEM, *n* = 8). ^*∗*^ *P* < 0.05 in comparison with control diabetic group.

We evaluated the effect of induction of diabetes on oxidative stress indices such as MDA and TAC. The MDA increased significantly while the TAC decreased significantly after induction of diabetes. Administration of extract causes a decrease in MDA (in all doses of extract, *P* < 0.01) and increase in TAC (in all doses of extract, *P* < 0.01) (Figures 2 and 3).

Induction of diabetes leads to significantly increased ALT, AST, and GGT activities as well as total bilirubin level in comparison to control groups (Table 3). After administration of* Peganum harmala* extract (in doses of 60 and 120 mg/kg), the mean values of hepatic functional indices such as ALT, AST, GGT, and bilirubin decreased significantly (*P* < 0.01) to normal range in dose dependent manner.

## 4. Discussion

This study was performed to find out the effect of oral administration of the hydroalcoholic extract of* Peganum harmala* seeds for 4 weeks on plasma glucose and lipid profile in diabetic male rats. Streptozotocin-induced diabetic rats showed significant increase in blood glucose as well as changes in lipid profile in comparison with normal rats. Our findings revealed that the hydroalcoholic extract of* Peganum harmala* seeds for 4 weeks significantly decreased the levels of glucose, triglyceride, cholesterol, and LDL-c and increased the level of HDL-c in diabetic rats. Singh et al. [7] showed that ethanolic extract of* Peganum harmala *seed significantly decreased (*P* < 0.001) blood glucose level in normal and diabetic rats at doses of 150 and 250 mg/kg of body weight.

*Peganum harmala *has been traditionally used to treat diabetes in folk medicine of some parts of the world [22]. It has been shown that methanolic extract of* Peganum harmala* has anticoccidial effects against* Eimeria tenella* [23]. Ethanol extract of* Peganum harmala* has been shown to be efficient in the treatment of cutaneous leishmaniasis [24]. There is an inverse association between dietary intakes of flavonoids and risk of cancer [25].

Flavonoids are naturally occurring phenolic compounds (i.e., flavonols, flavones, flavanones, flavan-3-ols, and anthocyanins) that are distributed in plants. They comprise wide range of biological activity such as antidiabetic, anticancer, antioxidant, and antimicrobial [26–32].

In human, dietary consumption of high anthocyanins and anthocyanin-rich fruit, particularly blueberries, decreased the risk of T2DM [29]. Animal studies have shown that anthocyanin improves glucose metabolism, insulin resistance, and *β*-cell dysfunction via GLUT4 regulation [26, 27, 33, 34].

Antidiabetic effects of* Peganum harmala *extract may be due to antioxidant, enzyme inhibition, and receptor agonist or antagonist activity or through novel mechanisms yet to be clarified. One of the possible explanation mechanisms for hypoglycemic action of hydroalcoholic extract of* Peganum harmala* seed may be due to enhanced insulin secretion from residual pancreatic *β*-cells from diabetic rats. Our data showed that* Peganum harmala* can decrease HbA_1C_ in diabetic rats, which emphasizes the hypoglycemic effect of extract. Treatment of nondiabetic rats with* Peganum harmala* did not have any effect on glucose and HbA_1C_ level.

Hyperlipidemia is one of well-known complications of diabetes. Helpful effects of* Peganum harmala* on lipid profile also may relate to effect on carbohydrate metabolism or modification of hepatic enzymes or decrease in lipid peroxidation and oxidative stress. Antioxidants can reduce the oxidative stress and consequently ameliorate the progress of stress related diseases. This study showed that MDA level decreased and TAC increased after treatment with* Peganum harmala* in diabetic rats. The antioxidant activity of the extract could decrease the rate of LDL oxidation. Berrougui et al. [35] found that* Peganum harmala* extract contained harmaline and harmine alkaloids. They showed that these compounds had significant free radical scavenging capacity and inhibited lipid peroxidation of LDLs.

Some of the harmful effects of diabetes may relate to hepatic dysfunction and may be corrected after use of* Peganum harmala* extract in diabetic rats. Our data revealed that liver function tests such as ALT, AST, GGT, and bilirubin change after diabetes induction and are modified after treatment which indicate that* Peganum harmala *extract possesses liver damage recovering effects.

It has been shown that aqueous extracts of* Peganum harmala* at dose of 300 mg/kg of body weight for 60 days might have adverse effects on the processes of spermatogenesis via direct or indirect effects on somniferous tubules and/or the pituitary testicular axis [36]. Oral administration of aqueous extract of* Peganum harmala* for six times a week at doses of 1, 1.35, and 2 g/kg during 3-month period increased transaminases in rats [37]. Histological findings revealed liver degeneration and spongiform changes in the central nervous system in rats treated with 2 g/kg dose but not at the therapeutic dose of 1 g/kg.

One limitation of the current study is that we did not perform histological evaluation of organs and tissues of* Peganum harmala* treated rats. The other limitation is that we did not determine the insulin level and pancreatic islet *β*-cell recovering effect of* Peganum harmala *extract.

## 5. Conclusion

Taken together, our findings indicate that hydroalcoholic extract of* Peganum harmala* exhibits antidiabetic and hypolipidemic activities in streptozotocin-induced diabetic male rats. Further studies are needed to establish the components of the extract and the mechanism(s) by which the extract utilizes its effects.

## Figures and Tables

**Table 1:** The mean of body weight (g) and daily food (g) and water intake (mL) per animal in all groups (data are shown as mean ± SD, *n* = 8).

Groups	Initial weight (g)	Final weight (g)	Food intake (g)	Water intake (mL)
Intact	229.6 ± 16.9	265 ± 17	17.25 ± 1.2	39.4 ± 3.9
Control + 30 mg/kg extract	228.8 ± 13.9	263.6 ± 18	17.4 ± 1.6	41.8 ± 5.9
Control + 60 mg/kg extract	223.1 ± 21.4	258.5 ± 24.8	16.4 ± 1.6	41.8 ± 5.7
Control + 120 mg/kg extract	229.8 ± 18.2	255.3 ± 20.1	15.6 ± 1.3	42.4 ± 7.1
Diabetic	237 ± 7.5	194.5 ± 15.2^*∗*^	35.9 ± 2.8^*∗*^	141.6 ± 26.8^*∗*^
Diabetic + 30 mg/kg extract	233.9 ± 10.4	189.1 ± 20.7	33.3 ± 2.9	143.7 ± 29.4
Diabetic + 60 mg/kg extract	234 ± 9.5	188.6 ± 19.9	35.1 ± 2.4	135.5 ± 25.2
Diabetic + 120 mg/kg extract	220.5 ± 11.4	182.8 ± 8.4	30.2 ± 3.6	119.0 ± 11.6

^*∗*^Statistically significant.

**Table 2:** The mean values of fasting glucose and lipids (mg/dL) profile in serum of all groups (data shown as mean ± SD, *n* = 8).

Groups	FBG (mg/dL)	Triglyceride (mg/dL)	Total cholesterol (mg/dL)	HDL-c (mg/dL)	LDL-c (mg/dL)
Intact	99.4 ± 17.8	67 ± 8.8	66.5 ± 6.7	31.3 ± 3.2	23.1 ± 4.8
Control + 30 mg/kg extract	85.9 ± 10	72.5 ± 18.9	68.8 ± 7.2	32.4 ± 4.1	22 ± 2.7
Control + 60 mg/kg extract	85.0 ± 18.0	50.8 ± 13.5	64 ± 12.4	31.5 ± 6.3	22.8 ± 7.3
Control + 120 mg/kg extract	99.6 ± 15.4	68.1 ± 20.1	67.4 ± 8	31.5 ± 4.2	22.4 ± 4.2
Diabetic	524.7 ± 75.6^*∗*^	91 ± 8.1^*∗*^	95.7 ± 4.2^*∗*^	25.6 ± 4.8^*∗*^	43.9 ± 12.8^*∗*^
Diabetic + 30 mg/kg extract	370.0 ± 111.7	82.2 ± 10.7	80.6 ± 9.7	35.1 ± 4.9	32.9 ± 6.8
Diabetic + 60 mg/kg extract	311.5 ± 59.5	70 ± 12.5	77.4 ± 14.4	34.5 ± 7.2	26.3 ± 6.6
Diabetic + 120 mg/kg extract	282.3 ± 45.3	69 ± 5.4	73 ± 6.2	45 ± 2.1	28.5 ± 3.1

^*∗*^Statistically significant.

**Table 3:** Mean values of AST, ALT, GGT, and bilirubin in all groups (data shown as mean ± SD, *n* = 8, ^*∗*^ *P* < 0.05 compared to diabetic group, and ^*∗∗*^ *P* < 0.01 compared to control group).

Groups	AST (IU/L)	ALT (IU/L)	GGT (IU/L)	Bilirubin (mg/dL)
Intact	146.25 ± 29.4	58 ± 13	22.1 ± 6.3	0.37 ± 0.02
Control + 30 mg/kg extract	157 ± 48.3	60 ± 10	22.6 ± 6.9	0.38 ± 0.01
Control + 60 mg/kg extract	156.4 ± 32.2	57 ± 8	22 ± 2.4	0.37 ± 0.01
Control + 120 mg/kg extract	141.2 ± 28.8	65 ± 5.5	21.5 ± 3.9	0.39 ± 0.02
Diabetic	383.8 ± 134^*∗∗*^	84.2 ± 3.9^*∗∗*^	36.1 ± 4.6^*∗∗*^	0.53 ± 0.08^*∗∗*^
Diabetic + 30 mg/kg extract	317.5 ± 53.2	75.6 ± 3.2	27.8 ± 2.8^*∗*^	0.4 ± 0.06^*∗*^
Diabetic + 60 mg/kg extract	249 ± 64.8^*∗*^	72 ± 5.3^*∗*^	25.7 ± 4.5^*∗*^	0.4 ± 0.03^*∗*^
Diabetic + 120 mg/kg extract	177.3 ± 34^*∗*^	71 ± 11.2^*∗*^	26.6 ± 3.4^*∗*^	0.41 ± 0.03^*∗*^
